# Evaluation of cardiovascular risk in adults with type 1 diabetes: poor concordance between the 2019 ESC risk classification and 10-year cardiovascular risk prediction according to the Steno Type 1 Risk Engine

**DOI:** 10.1186/s12933-020-01137-x

**Published:** 2020-10-03

**Authors:** Nicola Tecce, Maria Masulli, Roberta Lupoli, Giuseppe Della Pepa, Lutgarda Bozzetto, Luisa Palmisano, Angela Albarosa Rivellese, Gabriele Riccardi, Brunella Capaldo

**Affiliations:** 1 Department of Clinical Medicine and Surgery, Federico II University, Via S. Pansini 5, 80131 Naples, Italy; 2 Department of Molecular Medicine and Medical Biotechnology, Federico II University, Naples, Italy

**Keywords:** Type 1 diabetes, Cardiovascular risk, STENO Type 1 risk engine, ESC guidelines

## Abstract

**Background:**

Patients with type 1 diabetes (T1D) have higher mortality risk compared to the general population; this is largely due to increased rates of cardiovascular disease (CVD). As accurate CVD risk stratification is essential for an appropriate preventive strategy, we aimed to evaluate the concordance between 2019 European Society of Cardiology (ESC) CVD risk classification and the 10-year CVD risk prediction according to the Steno Type 1 Risk Engine (ST1RE) in adults with T1D.

**Methods:**

A cohort of 575 adults with T1D (272F/303M, mean age 36 ± 12 years) were studied. Patients were stratified in different CVD risk categories according to ESC criteria and the 10-year CVD risk prediction was estimated with ST1RE within each category.

**Results:**

Men had higher BMI, WC, SBP than women, while no difference was found in HbA1c levels between genders. According to the ESC classification, 92.5% of patients aged < 35 years and 100% of patients ≥ 35 years were at very high/high risk. Conversely, using ST1RE to predict the 10-year CVD risk within each ESC category, among patients at very high risk according to ESC, almost all (99%) had a moderate CVD risk according to ST1RE if age < 35 years; among patients aged ≥35 years, the majority (59.1%) was at moderate risk and only 12% had a predicted very high risk by ST1RE. The presence of target organ damage or three o more CV risk factors, or early onset T1D of long duration (> 20 years) alone identified few patients (< 30%) among those aged ≥35 years, who were at very high risk according to ESC, in whom this condition was confirmed by ST1RE; conversely, the coexistence of two or more of these criteria identified about half of the patients at high/very high risk also according to this predicting algorithm. When only patients aged ≥ 50 years were considered, there was greater concordance between ESC classification and ST1RE prediction, since as many as 78% of those at high/very high risk according to ESC were confirmed as such also by ST1RE.

**Conclusions:**

Using ESC criteria, a large proportion (45%) of T1D patients without CVD are classified at very high CVD risk; however, among them, none of those < 35 years and only 12% of those ≥ 35 years could be confirmed at very high CVD risk by the ST1RE predicting algorithm. More studies are needed to characterize the clinical and metabolic features of T1D patients that identify those at very high CVD risk, in whom a very aggressive cardioprotective treatment would be justified.

## Background

Worldwide estimates of numbers of individuals with Type 1 diabetes (T1D) continue to increase [1]. Among other reasons, this is particularly worrisome since adults with T1D have an almost threefold higher mortality compared to the general population, largely due to premature cardiovascular disease (CVD) [2–7]. In this respect, even in children and young adults with T1D, there is evidence of cardiac and vascular dysfunction, as supported by the presence of abnormal global myocardial function, stiffening of large arteries, and early atherosclerosis [8].

Hyperglycemia plays an important role in the onset and progression of vascular damage and, as demonstrated by DCCT/EDIC study, improving glycemic control substantially reduces the risk of microvascular complications and CVD [9]. However, according to more recent data from the Swedish National Diabetes Register, patients with T1D and a glycated hemoglobin level of 6.9% or lower still have a twofold risk of death compared to matched controls [2]. These findings, together with the evidence from randomized clinical trials (RCTs) [9] support the concept that, in addition to glycemic control, other factors concur to increase CVD incidence in these patients [9–12]. In fact, the risk of ischemic and hemorrhagic stroke rises linearly with blood pressure levels in individuals with T1D; this is already evident at values below the current treatment goals of 130/80 mmHg [13]. Moreover, the presence and severity of microvascular complications contribute to increase the risk of all-cause mortality and CVD outcomes in these patients [14]. As to the effects of pharmacological interventions on CV risk factors, at odds with type 2 diabetes (T2D), only very few RCTs have assessed the impact of this strategy on the incidence of CVD outcomes in T1D patients—well characterized for their clinical and metabolic features—in a primary prevention setting [15, 16]. Thus, at present, the stratification of CVD risk in T1D patients is mainly based on observational data [10].

In 2019, updated guidelines for the management and prevention of CVD risk in individuals with diabetes have been issued by the European Society of Cardiology (ESC) [17]. According to ESC criteria, most T1D patients—in the absence of previous CVD—are at high/very high risk [17]. This has a major impact on the clinical care of these patients, particularly younger ones, since it implies not only very ambitious targets for LDL-cholesterol, often achievable only with aggressive treatment strategies, including the very expensive therapies now available, but also more stringent blood pressure control and the use of antiplatelet agents [18]. Therefore, it would be desirable to identify relevant features of T1D patients that characterize the presence of higher CVD risk to identify those who would qualify for a more aggressive treatment of CVD risk factors.

To this aim, risk scores and other CVD biomarkers have been developed for the risk stratification of T1D patients [19–25]. The American Diabetes Association (ADA) strongly supports their utilization for the assessment of the 10-year risk of a first CVD event to help guide treatment strategy [26]. Among these, a recently published CVD prediction model—the Steno Type 1 Risk Engine (ST1RE)—has shown a high performance in predicting 10-year CVD events in a cohort of 4996 T1D adults without previous CVD events [23], and also in identifying T1D patients with preclinical atherosclerosis [27, 28].

Against this background, the aim of the present study was to evaluate the concordance between 2019 ESC CVD risk classification and 10-year CVD risk predicted by ST1RE in a cohort of unselected T1D patients without previous CVD. We focused particularly on the very high risk category because of the strict recommended targets for CVD risk factors, requiring high intensity cardio-protective therapy.

## Methods

### Study design and population

This is an observational retrospective, single-center study in a cohort of five hundred seventy-five individuals with T1D (272F/303M, age range 18–74 years). All participants attended the Outpatient Diabetes Clinic of Federico II University Hospital, Naples (Italy) from January 2015 to December 2018; they underwent yearly evaluation for the routine screening of chronic complications. The medical records of each patient’s most recent visit were reviewed to collect clinical and biochemical variables: body mass index (BMI), waist circumference (WC), systolic blood pressure (SBP), diastolic blood pressure (DBP), lipid profile (total cholesterol, HDL cholesterol, triglycerides), duration of diabetes, insulin therapy, smoking status, physical activity, presence of target organ damage (proteinuria, renal impairment defined as eGFR < 30 ml/min/1.73 m^2^, left ventricular hypertrophy, or retinopathy), presence of chronic diabetic complications, comorbidities, other autoimmune diseases (autoimmune thyroiditis, celiac disease, Addison disease, systemic lupus erythematosus, vitiligo) and medication use.

### Measurements and definitions

Plasma concentrations of glucose and lipids were measured by standard methods; glycated hemoglobin (HbA1c) by High-performance liquid chromatography (HPLC); albumin concentration in spot urine by immunonephelometry, and plasma and urine creatinine by the modified Jaffé reaction using an autoanalyzer (Pentra 400, Horiba ABX Diagnostics). All biochemical analyses were centralized and were performed under appropriate quality control.

BMI was calculated as weight in kg/height in m^2^. Low density lipoprotein (LDL)—cholesterol was calculated by Friedwald’s formula. eGFR was calculated using the CKD-EPI formula. Smoking status was defined as smoking one or more cigarettes per day. Physical exercise was defined as a dichotomic variable (1 = if the patient exercised more than 30 min per day; 0 = if the patient exercised less than 30 min per day). Hypertension was diagnosed as SBP ≥ 140 mmHg or DBP ≥ 90 mmHg and/or use of antihypertensive drugs [29]. Hypercholesterolemia was defined as LDL cholesterol > 100 mg/dl or use of cholesterol-lowering drugs.

### Cardiovascular risk assessment

CVD risk categories were defined according to 2019 ESC guidelines, which classify T1D patients into 3 categories: (1) very high CVD risk (≥ 10% 10-year risk of fatal CVD events), which includes patients who have a previous CVD, or with target organ damage, or three or more major CVD risk factors, or early-onset T1D of long duration (> 20 years); (2) high risk (5–9% 10-year risk of fatal CVD), which comprises all patients not included in the very high or the moderate risk category, and (3) moderate risk (3–4% 10-year risk of fatal CVD events), which includes young patients (aged < 35 years) with T1D duration < 10 years without other CVD risk factors. To convert the risk of fatal to that of total (fatal + non fatal) CVD, the former was multiplied by 3 in men and by 4 in women, as suggested by ESC [18].

To evaluate the concordance between the ESC risk classification and the ST1RE prediction model, we analyzed the very-high risk category first as a whole and then separately for each subcategory according to the presence of: (a) target organ damage (proteinuria, eGFR < 30 mL/min/1.73 m^2^, left ventricular hypertrophy, or retinopathy); (b) three or more risk factors (age > 35 years, hypertension, hypercholesterolemia, smoking, obesity); (c) early-onset T1D of long duration (> 20 years) or a combination of these. Within each risk category, the prediction of the 10-year risk of a first fatal/non-fatal CVD event was performed using ST1RE, which employs a composite end point including ischemic heart disease, ischemic stroke, heart failure, and peripheral artery disease. In addition to traditional CVD risk factors such as age, sex, SBP, LDL-cholesterol, eGFR, smoking, ST1RE considers some variables more related to diabetes status, such as diabetes duration, HbA1c, albuminuria, and physical exercise [23]. For our analyses, this model was applied in 532 patients since 22 had previous CVD and for 21 patients some data were missing.

### Assessment of microvascular complications

Microalbuminuria was defined as urinary albumin excretion rate (UAE) > 30 mg/L in at least three 24 h-urine samples obtained at about three months’ intervals in the year preceding recruitment. eGFR was estimated using the CKD-EPI formula. Diabetic nephropathy was defined as two or more positive microalbuminuria test results (UAE of 3–30 mg/mmol on spot urine) within 6 months. Autonomic neuropathy was evaluated by means of standardized cardiovascular reflex tests: parasympathetic function with beat-to-beat variation test, and sympathetic function with the blood pressure response to standing (a fall in systolic blood pressure of at least 30 mmHg when the patient moved from the sitting to the standing position was considered diagnostic). Peripheral neuropathy was diagnosed according to the “Michigan Neuropathy Screening Instrument” based on the clinical evaluation of bilateral vibration perception test, ankle reflex assessments and tactile perception with the Semmes–Weinstein monofilament.

### Statistical analysis

Data are expressed as mean ± standard deviation (SD) or proportion (%). To compare continuous variables, an independent samples t test was performed. The Chi square test was used to analyze categorical data. A two-side *p* value < 0.05 was considered significant. The Statistical analysis was performed using SPSS v.22 (SPSS, Inc. Chicago, IL USA).

## Results

The main clinical and metabolic characteristics of the study population are shown in Table 1. Men had significantly higher values of BMI, WC, SBP, DBP than women (p < 0.01) while there was no difference in HbA1c levels between the two genders. Total cholesterol and HDL-cholesterol levels were higher in women (p = 0.04 and p < 0.0001, respectively) while triglycerides were significantly higher in men (p < 0.0001). One-third of the participants were on continuous subcutaneous insulin infusion (CSII), with a significantly higher prevalence among women (p = 0.003). As expected, the presence of other autoimmune diseases was higher in women than in men (44.9% vs. 25.4%, p < 0.0001). As to the prevalence of microvascular complications, only retinopathy was significantly higher in men than in women (22.1% vs 15.2%, p = 0.038). A history of CVD was present in 3.8% of the study cohort, without differences between genders.

**Table 1:** General characteristics of the study population

	Total n = 575	Females n = 272	Males n = 303	P
Age (years)	36 ± 12	36 ± 13	35.6 ± 12.1	0.789
Diabetes duration (years)	19 ± 11	19.1 ± 10.5	18.8 ± 10.9	0.739
Early onset T1D (1–10 years of age) (%)	31.3	32.4	30.4	0.653
Age of T1D onset (years)	16.8 ± 10.8	16.7 ± 11.1	17 ± 10.4	0.712
Waist circumference (cm)	86.0 ± 11.8	82.4 ± 11.3	89.3 ± 11.2	< 0.0001
BMI (kg/m^2^)	25.1 ± 3.6	24.7 ± 3.8	25.4 ± 3.3	0.018
HbA1c (%)	7.7 ± 1.2	7.9 ± 1.2	7.7 ± 1.22	0.064
SBP (mmHg)	121 ± 16	118 ± 17	123 ± 14	< 0.0001
DBP (mmHg)	75 ± 10	73 ± 11	76 ± 9	0.001
Total cholesterol (mg/dl)	178 ± 34	181.5 ± 35.7	175.6 ± 33	0.040
LDL-cholesterol (mg/dl)	101 ± 29	100 ± 28.1	101.5 ± 29.5	0.468
HDL-cholesterol (mg/dl)	62 ± 16	68 ± 17.3	56.9 ± 13.9	< 0.0001
Triglycerides (mg/dl)	79 ± 52	71.2 ± 36.7	86.5 ± 62.3	< 0.0001
eGFR (CKD-EPI) (ml/min/1.73 m^2^)	101.8 ± 18	100.7 ± 18.6	102.8 ± 17.5	0.168
Other autoimmune diseases (%)	35	44.9	25.4	< 0.0001
CSII (%)	32.5	38.7	26.9	0.003
Microvascular complications (%)	27.7	26.8	28.4	0.679
Nephropathy (%)	9.7	9.4	10.1	0.778
Retinopathy (%)	18.8	15.2	22.1	0.038
Neuropathy (%)	10.4	10.9	10.07	0.727
Previous CVD (%)	3.8	3.3	4.3	0.540
Hypertension (%)	29.4	25.4	33	0.050
Hypercholesterolemia (%)	53	49.6	56.1	0.132

Data are expressed as Mean ± standard deviation or as a percentage

*SBP* systolic blood pressure, *DBP* diastolic blood pressure, *CVD* Cardiovascular disease (Ischemic heart disease, stroke, peripheral arteriopathy), *eGFR* estimated glomerular filtration rate, *CSII* continuous subcutaneous insulin infusion

**Fig. 1:**
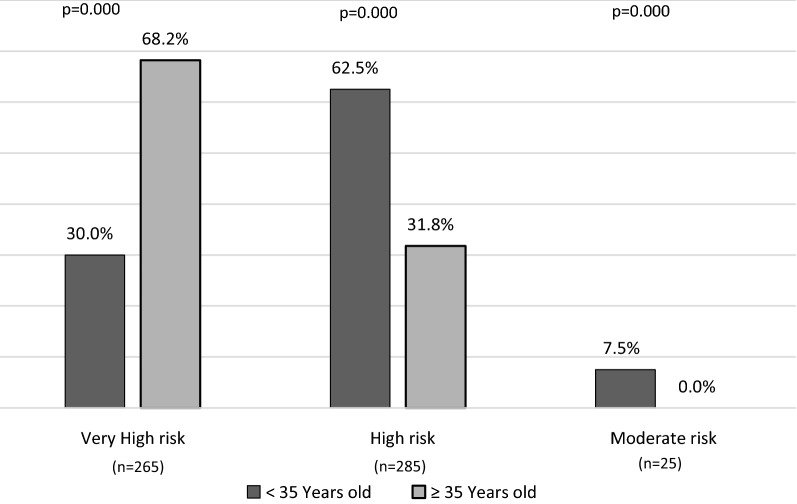
Cardiovascular risk classification according to 2019 ESC criteria in patients aged < 35 or ≥ 35 years (n = 575). Total (fatal + non fatal) CVD risk was estimated in the total population, stratified by age (< 35 or ≥ 35 years). CVD risk categories were defined according to 2019 ESC guidelines. Very high CVD risk category included T1D patients with a history of CVD, or target organ damage (proteinuria, eGFR < 30 ml/min/1.73 m^2^, left ventricular hypertrophy, or retinopathy), or three or more major CVD risk factors (age > 35 years, hypertension, hypercholesterolemia, smoking, obesity), or early-onset T1D of long duration (> 20 years); high risk category included all patients not included in the very high or the moderate risk category; moderate risk group included young patients (aged < 35 years) with T1D duration < 10 years without other risk factors

Figure 1 shows the CVD risk classification of patients aged < 35 or ≥ 35 years, according to 2019 ESC guidelines. Among patients aged < 35 years, 30% belonged to the very high risk category, the large majority (62.5%) to the high risk category, and only as few as 7.5% to the moderate risk category. Among patients aged ≥ 35 years, 68.2% belonged to the very high risk category, 31.8% to the high risk category and none was classified as being at moderate risk.

Table 2 reports the estimated 10-year CVD risk using the ST1RE prediction model within each category of risk defined by the 2019 ESC criteria. Among patients aged < 35 years, all those in the moderate risk group were confirmed as such by the Steno predictive algorithm; however, among those classified in the high risk category by ESC, 100% were also at moderate risk according to the ST1RE prediction algorithm. Noteworthy, none of the 96 patients classified as being at very high risk by ESC were confirmed as such by ST1RE, only 1 was at high risk and all the others were at moderate risk.

**Table 2:** 10-year CVD risk prediction according to Steno type 1 Risk Engine (ST1RE) in a cohort of adults aged < 35 or ≥ 35 years in whom the CVD risk was classified according to 2019 ESC criteria (n = 532)

CVD risk classification according to 2019 ESC guidelines	10-year CVD risk stratification predicted according to ST1RE		
	Very high risk	High risk	Moderate risk
**Patients < 35 years (n = 316)**			
Very high risk n = 96	0 (0)	1 (1)	95 (99)
Target organ damage n = 26	0 (0)	0 (0)	26 (100)
Three or more risk factors n = 5	0 (0)	0 (0)	5 (100)
Early onset T1D of long duration (> 20 years) n = 50	0 (0)	0 (0)	50 (100)
Two or more among the above criteria n = 15	0 (0)	1 (6.7)	14 (93.3)
High risk n = 197	0 (0)	0 (0)	197 (100)
Moderate risk n = 23	0 (0)	0 (0)	23 (100)
**Patients ≥ 35 years (n = 216)**			
Very high risk n = 142	17 (12)	41 (28.9)	84 (59.1)
Target organ damage n = 23	2 (8.7)	4 (17.4)	17 (73.9)
Three or more risk factors n = 49	4 (8.2)	14 (28.6)	31 (63.2)
Early onset T1D of long duration (> 20 years) n = 10	0 (0)	3 (30)	7 (70)
Two or more among the above criteria n = 60	11 (18.3)	20 (33.3)	29 (48.4)
High risk n = 74	0 (0)	6 (8.1)	68 (91.9)
Moderate risk n = 0	0 (0)	0 (0)	0 (0)

Data are expressed as number (n) and percentage (%). The analysis was performed in 532 patients since 22 patients had a previous CVD and 20 patients had some missing data

Target organ damage included proteinuria, eGFR < 30 ml/min/1.73 m^2^, left ventricular hypertrophy, or retinopathy

CVD risk factors were: age > 35 years, hypertension, hypercholesterolemia, smoking, obesity

Among patients aged ≥ 35 years, none qualified as being at moderate risk according to ESC. In the high risk group according to ESC, only 8.1% was confirmed at high risk and as many as 92% had in fact a moderate risk according to ST1RE (Table 2). In the very high risk group according to ESC criteria, 12% were predicted to be at very high risk, 28.9% at high risk and 59.1% at moderate risk according to the ST1RE.

Focusing on the ESC subcategories of very high risk patients aged ≥ 35 years, 8.7% of patients classified as being at very high risk because of the presence of target organ damage and 8.2% of those with three or more CV risk factors were confirmed to be at very high risk according to ST1RE, while the large majority (60–70%) of them was at moderate risk. Remarkably, among patients classified at very high risk because of early onset T1D of long duration (> 20 years), none was confirmed to be at very high risk, 30% was at high risk whereas the vast majority (70%) had a predicted moderate risk according to ST1RE. A much larger proportion of patients (52%) in the subcategory with two or more ESC criteria for the definition of a very high risk was confirmed to be at high or very high risk by ST1RE (Table 2).

**Fig. 2:**
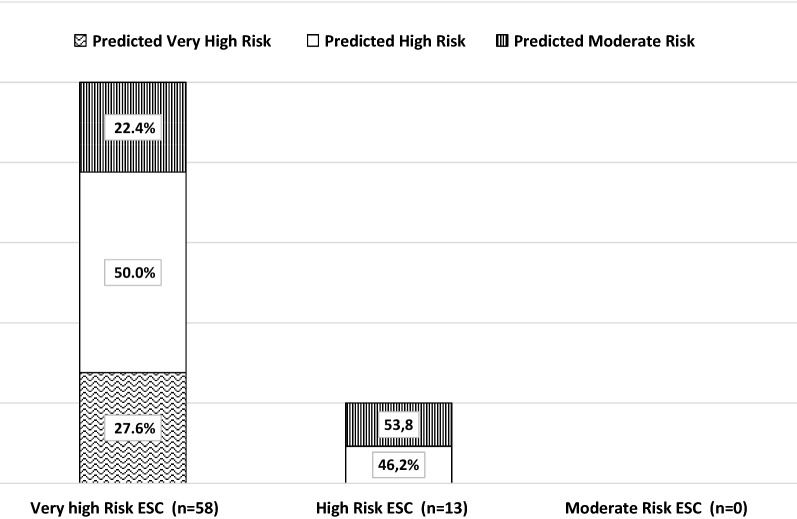
Prediction of CVD events using the Steno type 1 Risk Engine (ST1RE) in a cohort of adults aged ≥ 50 years classified according to 2019 ESC criteria (n = 71). The population over 50 years was classified according to 2019 ESC guidelines into three groups: very high risk, high risk and moderate risk. 10-year CVD risk prediction was estimated with ST1RE

Since from the analysis reported in Fig. 1 and Table 2, age appears to have a relevant impact on CVD risk, we decided to perform CVD risk prediction by ST1RE in patients classified according to 2019 ESC, limiting the analysis to those aged ≥ 50 years. As shown in Fig. 2, the ESC risk classification performed much better in this age group, since 77% of patients at high/very high risk according to ESC were confirmed as such also by the ST1RE prediction score.

## Discussion

### Evaluating CVD risk in T1D patients

Recently, ESC—in collaboration with EASD—has issued the 2019 Guidelines on Diabetes, Pre-Diabetes and Cardiovascular Diseases, where CVD risk classification is proposed also for patients with T1D [17]. Noteworthy, however, CVD risk stratification for these patients has been largely based on observational studies [10] and never validated in relation to measured CVD events. In fact, there are very few controlled studies conducted in T1D individuals on the effect of treatment on CVD outcomes [31] or risks factors [15, 16]. In the EMERALD (Effects of Metformin on CardiovasculaR function in AdoLescents with Type 1 diabetes) trial, metformin therapy as add-on to insulin improved insulin resistance and vascular health [16]. In contrast, the Adolescent Type 1 Diabetes Cardio-Renal Intervention Trial (AdDIT study) showed that the use of an ACE inhibitor and a statin did not change albumin—to-creatinine ratio (ACR), carotid intima-media thickness or other CVD markers in adolescents (10–16 years) with T1D, over a median period of 2.6 years [15]. Although these studies indicate some cardiovascular benefits of intervention on CV risk factors in primary prevention, they do not provide reliable information on how to identify T1D patients for whom the implementation of these treatments would be justified on the basis of a risk/benefit analysis. In fact, previous experience with T2D patients is of limited usefulness since different patterns of risk factors predict CVD in type 1 and type 2 diabetes [30–33]. Based on these premises, the American Diabetes Association (ADA) advices the implementation of predictive algorithms as a useful tool for T1D patients-specific CVD risk estimation [27]; among them, ST1RE represents one of the best options [23]. This is a model to predict 5- and 10-year CVD risk in adults with T1D, validated in the T1D population of the Funen Diabetes Database in Denmark; in addition, it was able to predict preclinical atherosclerosis in Spanish adults with T1D [28, 29]. The Steno T1D risk score was able to identify individuals with subclinical atherosclerosis and high CVD risk also in a T1D population in Italy. However, the absolute risk was significantly overestimated despite the rather low number of events [34].

As far as we know, the present study is the first to evaluate CVD risk according to a prediction model (ST1RE) in an unselected real-life sample of adults with T1D without previous CVD, classified in relation to their presumed CVD risk according to 2019 ESC guidelines.

### Concordance between 2019 ESC risk classification and 10-year prediction of CVD risk by ST1RE

The results of this study show poor concordance between the 2019 ESC risk classification in T1D patients and the predicted 10-year risk of a CVD event, as estimated by ST1RE. In fact, according to the ESC classification, as many as 45% of our patients without previous CVD events fell in the very high CVD risk category, but, according to ST1RE, only 12% of them had a predicted very high risk of CVD events in the subsequent 10 years. Notably, our patients were young (mean age 36 ± 12 years) and in a reasonable blood glucose control, had relatively well controlled blood pressure and plasma lipid levels, and a low prevalence of microvascular complications.

The lack of concordance was particularly large in subjects aged < 35 years: it is remarkable that almost all patients (99%) in this age group, classified in the very high risk category according to ESC, were only at moderate risk according to ST1RE. However, also among patients aged ≥ 35 years, concordance was poor since the large majority (59%) of those classified at very high risk according to ESC criteria was only at moderate risk according to ST1RE prediction, 29% were at high risk and only as few as 12% were confirmed to be at very high risk.

We used the age cut-off of 35 years as suggested by ESC guidelines; nevertheless, stratifying the population according to an arbitrary higher cut-off (50 years)—as suggested by ESC for type 2 diabetes patients—, the concordance between the ESC CVD risk classification and the ST1RE 10-year CVD risk prediction becomes much better; in fact, among patients aged ≥ 50 years, 78% of those classified in the very high risk category by ESC guidelines are confirmed to be at high/very high risk by the Steno algorithm (Fig. 2).

ESC guidelines largely emphasize early onset T1D of long duration as one of the major determinants of CVD risk events. In fact, they consider people with early onset T1D and > 20 years duration in the same risk category as patients with a previous CVD event. The CVD predictive value of early onset diabetes and long diabetes duration is based on several cohort and registry studies [7, 35, 36]. In T1D subjects of the Swedish National Diabetes Register [7], the early onset of diabetes (1-10 years of age) was associated with a significantly higher risk of CVD events compared to T1D onset between the ages of 26–30 years [7], and resulted in loss of 17.7 years of life in women and 14.2 years in men [7]. In an observational study of an Australian cohort of 1169 T1D patients, Pease et al. showed a J-shaped association between diabetes duration and CVD, with a threshold effect at approximately 20 years [35]. In our study, among patients classified by ESC at very high risk because of early-onset T1D of long duration (> 20 years), none had a very high predicted risk according to ST1RE. Although the reason for this inconsistency remain unclear, it is reasonable to hypothesize that early onset T1D of long duration increases the predictive power for CVD events when associated to poor glycemic control. To this regard, measurement of HbA1c needs, probably, to be integrated with the novel metrics of glycemic control that have recently been introduced in clinical practice [37]; among these, high glycemic variability has emerged as an important determinant of vascular damage [38]. Future studies are needed to establish whether the inclusion of this parameter will improve the prediction of CVD risk in T1D patients.

In our population, target organ damage, multiple CVD risk factors or early onset diabetes of duration > 20 years were not strong predictors of CVD events, if taken individually. On the contrary, the coexistence of two or more of these conditions in subjects ≥ 35 years, identified people at high/very high risk, who were confirmed as such also using ST1RE.

### Clinical implications

The assessment of CVD risk in T1D patients has important clinical implications, since different risk factors targets (especially LDL-cholesterol) and therapeutic strategies should be implemented in relation to CVD risk stratification [17]. Indeed, according to the ESC classification, around 45% of our cohort without previous CVD event (n = 238) should be aggressively treated with high-intensity statins and antiplatelet agents, because at very high CVD risk. However, if we consider at very high risk only those confirmed by ST1RE, the number of patients to be aggressively treated would be much lower (n = 17). Our findings are in line with the ADA statement that not all T1D patients should be considered at the same CVD risk level and that, while a moderate hypolipidemic approach is indicated in younger subjects in primary prevention, a high-intensity statin therapy is required only in those aged 40–75 years, with multiple CVD risk factors [27]. The implementation of strategies with a more aggressive treatment of patients at higher risk while using moderate intensity therapies in those at lower risk is essential to maximize the efficacy for each individual patient and minimize the costs for the healthcare system.

### Strengths and limitations

The main strengths of this study are: (1) the relatively large sample size of the cohort studied; (2) the adoption of standard diagnostic procedures, including physical examination, screening of chronic complications, central analysis of biochemical variables; (3) the evaluation of the predicted CVD risk performed by the ST1RE—a validated tool, specific for patients with T1D.

Some limitations should be acknowledged: firstly, the monocentric, observational study design and, secondly, the CVD risk evaluation was performed with a prediction model rather than by measuring hard endpoint (CVD events).

## Conclusions

Our study shows a poor concordance between ESC risk classification and 10-year CVD risk predicted by ST1RE, at least in young patients with T1D. A greater accuracy of ESC risk classification was demonstrated in patients ≥ 50 years of age. More studies are needed to validate predictive CVD risk algorithms versus event rates and to characterize clinical and metabolic features of T1D patients that identify those at very high CVD risk, in whom a very aggressive cardio-protective treatment would be justified.

## Data Availability

Data collected for this study can be shared and made available upon reasonable request to the corresponding author and subject to an approved proposal and data access agreement.

## Abbreviations

BMI: Body mass index
SBP: Blood pressure
DBP: Diastolic blood pressure
CVD: Cardiovascular disease
ASCVD: Atherosclerotic Cardiovascular Disease (Ischemic heart disease, stroke, peripheral arteriopathy)
eGFR: Estimated glomerular filtration rate
CSII: Continuous subcutaneous insulin infusion
